# Machine Learning in Gel-Based Additive Manufacturing: From Material Design to Process Optimization

**DOI:** 10.3390/gels11080582

**Published:** 2025-07-28

**Authors:** Zhizhou Zhang, Yaxin Wang, Weiguang Wang

**Affiliations:** 1 Department of Mechanical and Aerospace Engineering, School of Engineering, The University of Manchester, Manchester M13 9PL, UK; 2 Centre for the Cellular Microenvironment (CeMi), University of Glasgow, Glasgow G12 8QQ, UK; 3 Department of Mechanical Engineering, School of Engineering, University of Southampton, Southampton SO17 1BJ, UK

**Keywords:** machine learning, gels additive manufacturing, material design, process optimization

## Abstract

Machine learning is reshaping gel-based additive manufacturing by enabling accelerated material design and predictive process optimization. This review provides a comprehensive overview of recent progress in applying machine learning across gel formulation development, printability prediction, and real-time process control. The integration of algorithms such as neural networks, random forests, and support vector machines allows accurate modeling of gel properties, including rheology, elasticity, swelling, and viscoelasticity, from compositional and processing data. Advances in data-driven formulation and closed-loop robotics are moving gel printing from trial and error toward autonomous and efficient material discovery. Despite these advances, challenges remain regarding data sparsity, model robustness, and integration with commercial printing systems. The review results highlight the value of open-source datasets, standardized protocols, and robust validation practices to ensure reproducibility and reliability in both research and clinical environments. Looking ahead, combining multimodal sensing, generative design, and automated experimentation will further accelerate discoveries and enable new possibilities in tissue engineering, biomedical devices, soft robotics, and sustainable materials manufacturing.

## 1. Introduction

Gel materials have become fundamental to additive manufacturing in both biomedical [1,2,3] and engineering [4,5,6,7] applications. Their ability to hold large amounts of water while maintaining tunable mechanical properties makes them ideal for replicating native tissue environments [8,9]. These attributes enable applications ranging from scaffolds for tissue growth [10] and regenerative medicine [11] to controlled drug [12] release platforms and flexible wearables [13]. Despite the promise, traditional gel printing still relies heavily on trial-and-error methods. Formulation and printing parameter selection remain time-intensive and prone to variability. This bottleneck inhibits scalability and consistent fabrication outcomes [14].

Artificial intelligence and machine learning approaches are transforming the way materials are developed and processes are optimized [15,16,17]. Machine learning techniques [18,19,20,21,22,23,24,25,26], including decision trees, random forests, deep learning, and support vector machines, are being applied to predict the behavior of gels based on rheological and compositional inputs [27]. For instance, interpretable machine learning models have been used to explain how additives impact print fidelity using bulk material properties [28]. By linking formulation data to performance outcomes, AI enables an efficient search within vast compositional spaces, yielding faster formulation cycles and reduced experimental cost [29,30].

Nonetheless, challenges remain before such smart printing becomes mainstream. Data on gel systems are still limited in size and diversity and models often lack interpretability and robustness across new material spaces [31]. Translating AI methods to commercial hardware requires standardized interfaces and validation workflows that currently do not exist for most systems [32]. Furthermore, establishing trust in AI-recommended formulations in healthcare demands transparent machine learning frameworks backed by physical insight and regulatory validation [33,34].

**Figure 1:**
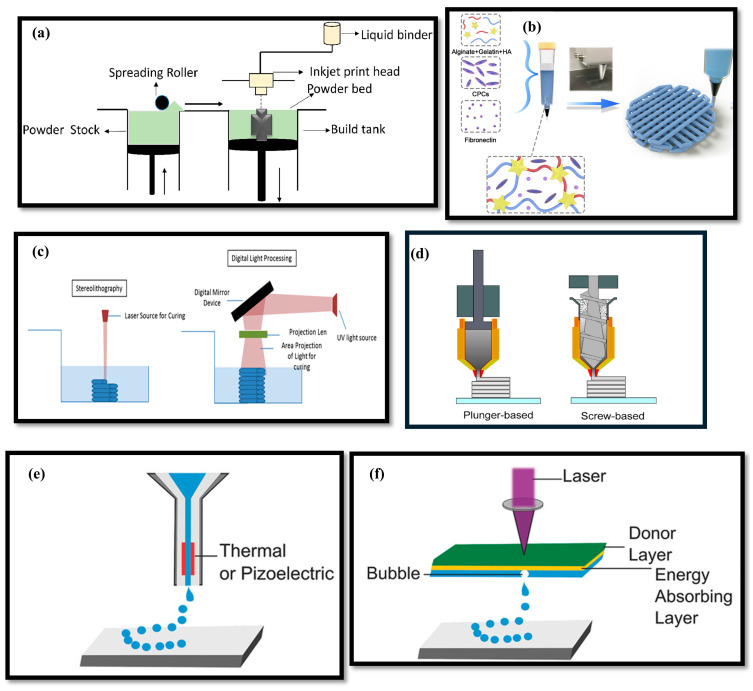
Schematics of gels printing: (**a**) binder jetting [36], (**b**) direct ink writing [37], (**c**) vat photopolymerization [38], including stereolithography (left) and digital light processing (right), (**d**) material extrusion [39], (**e**) inkjet jetting bioprinting [40], and (**f**) laser assisted bioprinting [40]. Panel a adapted from ref. [36], Elsevier, CC BY 4.0. Panel b adapted from ref. [37], Elsevier, CC BY-NC-ND 4.0. Panel c adapted from ref. [38], AccScience Publishing, CC BY-NC 4.0. Panel d adapted from ref. [39], MDPI, CC BY 4.0. Panels e and f adapted from ref. [40], Wiley, CC BY 4.0.

Gel-based additive manufacturing (AM) encompasses a variety of printing technologies, each with distinct mechanisms and achievable resolutions for fabricating soft, hydrated materials [35]. Figure 1 presents a comparative schematic of several leading gel AM methods: binder jetting, direct ink writing (DIW), vat photopolymerization (including stereolithography and digital light processing), material extrusion, inkjet bioprinting, and laser-assisted bioprinting. Each technique offers unique advantages in terms of resolution, material compatibility, and suitability for biomedical and engineering applications [36,37,38,39,40].

Binder jetting (Figure 1a) involves the selective deposition of a liquid binder onto a bed of powdered or gel precursor material [41,42]. The typical resolution for binder jetting ranges from about **50 to 100 μm** [43,44], determined mainly by the droplet size and the powder granularity [36,39,45]. While this approach allows the fabrication of complex and porous scaffolds, it often requires post-processing, such as debinding, sintering, and infiltration, to attain the desired mechanical integrity [46]. The use of machine learning has recently improved binder distribution and print fidelity within these constraints [47].

Direct ink writing (Figure 1b) is a versatile extrusion-based method where gel or hydrogel inks are deposited through a nozzle according to a programmed path [48]. Direct ink writing can achieve resolutions of 50 **μm** [49], governed by the nozzle diameter and ink rheology [37,50,51]. Machine learning models, particularly random forests and neural networks, have been used to optimize ink formulations for printability and shape retention at these fine scales [52,53,54,55]. Finer nozzles permit higher resolution but risk clogging or instability if ink rheology is not optimal [56].

Vat photopolymerization (Figure 1c), which includes stereolithography (SLA) and digital light processing (DLP), utilizes photosensitive gel precursors that are cured layer-by-layer with patterned light [57]. These technologies are noted for their high precision, with SLA typically reaching resolutions of <5 μm [58]; DLP can achieve a pixel size of 15 μm and can print with a layer thickness of 25 μm [59]. High resolution makes them particularly suitable for applications requiring smooth surfaces and intricate features, especially for gels [60].

Extrusion additive manufacturing (Figure 1d) extends a wider range of gel materials, which is the most common way for gel printing [61,62,63,64,65,66,67]. The typical resolution is about 500 μm [68], mainly determined by nozzle diameter and extrusion rate [69,70]. While not as fine as vat photopolymerization or DIW, material extrusion is ideal for creating larger constructs and can be efficiently optimized using machine learning for robust print fidelity.

Inkjet bioprinting (Figure 1e) uses pulsed droplets of gel-based bioinks, typically achieving a resolution in the range of 5–10 μm with 80 μm nozzle [71]. This high spatial precision is valuable for the patterned deposition of cells and biomolecules [72]. Machine learning methods have been successfully applied to control droplet formation [73], assess print quality [74], and enhance the uniformity of bioprinted structures [75].

Laser-assisted bioprinting (Figure 1f) uses focused laser pulses to propel minute quantities of gel precursors onto a substrate, with a typical achievable resolution of 30 to 60 μm [76,77]. The non-contact nature of this technique provides superior spatial accuracy and is particularly well-suited for fragile or sensitive biological samples [78]. Currently, no reports about machine learning algorithms support the optimization of laser-assisted bioprinting.

## 2. Fundamentals of Gel Additive Manufacturing

**Figure 2:**
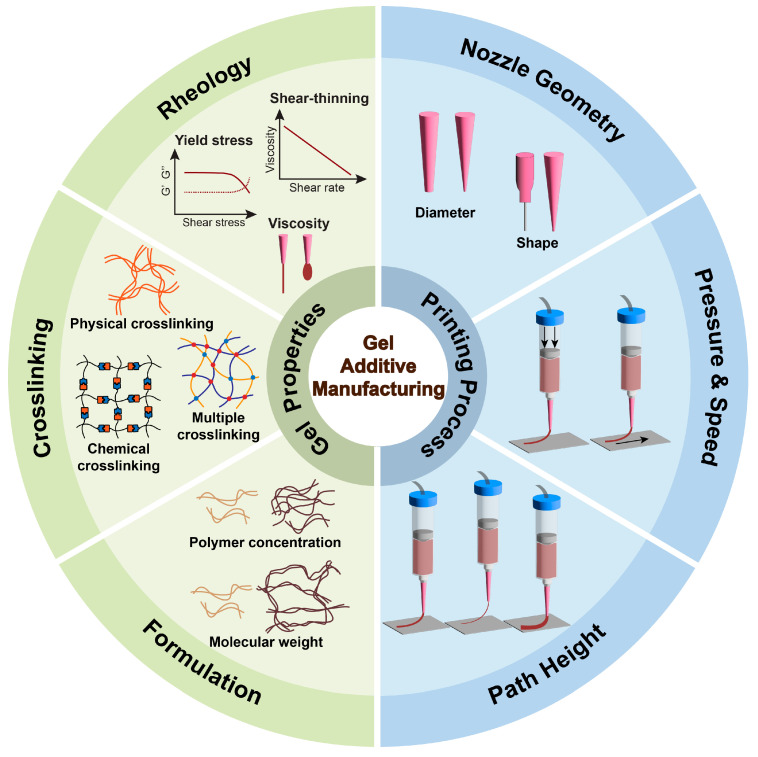
Interdependent material properties and process parameters in gel-based additive manufacturing.

Gel additive manufacturing enables the fabrication of soft hydrated materials and often biologically compatible structures with spatial precision [79]. The technique is distinguished by the use of gel precursors [80], where the materials display tunable behaviors and typically undergo a crosslinking process to solidify into stable three-dimensional (3D) structures. The effectiveness of gel additive manufacturing depends on a balance between gel properties and printing parameters [81,82]. In Figure 2, this schematic outlines the critical interdependencies between gel material properties—including viscosity, shear-thinning behavior, viscoelasticity, yield stress, and crosslinking strategies—and process parameters such as nozzle diameter, extrusion pressure, printing speed, and path height [51,83,84,85]. These factors collectively govern the success of gel-based additive manufacturing by influencing filament continuity, shape fidelity, and structural integrity. A deep understanding of these relationships provides the necessary foundation for applying machine learning techniques to optimize formulation design and printing conditions in a data-driven, predictive manner.

Rheological properties are crucial to gel printability [86,87]. Low viscosity can lead to the formation of undesirable droplets, causing uneven filament merging and cell sedimentation during printing [80,88]. Conversely, high viscosity allows the printing of continuous and stable filaments, but it increases shear stresses to encapsulated cells, reducing their viability [88]. Shear-thinning behavior, in which ink viscosity decreases with increasing stress, facilitates printing by reducing viscosity during extrusion without compromising cell viability [89]. The viscosity recovers rapidly post-deposition to prevent structural collapse. Viscoelasticity, characterized by storage (elastic) modulus G′ and loss (viscous) modulus G″, enables the gel inks to maintain shape fidelity during printing and exhibit elastic shape retention afterwards [90]. Yield stress, referring to the stress while the deformation of gel inks occurs, is also essential to printability. It limits the linear viscoelastic range and flow point of inks, leading to an increased stress to initiate the extrusion but also an enhanced stiffness and shape retention of the extruded filaments [80]. These rheological behaviors are highly correlated to the system of the gels such as the crosslinking mechanism, molecular weight, and polymer concentration [91,92].

Crosslinking strategies further modulate printability and other gel properties [85]. Natural materials such as gelatin and alginate [93] have been widely used to develop gel inks due to their physically crosslinkable ability under mild conditions (e.g., temperature and ions) without chemical modification [94,95]. Despite the attraction of physical crosslinking, its relatively slow gelation and weak mechanical stability post-printing limit its applications. Pre-crosslinking chemistries such as thermal and ionic crosslinking have been introduced to enhance this process [96,97,98]. Chemical modification is an alternative strategy to allow additional crosslinking and improve the printability of gel inks. For example, gelatin and hyaluronic acid have been modified by methacrylate and norbornene groups to allow in situ photocrosslinking [99]. The photocrosslink ability of gel inks enables the smooth extrusion of inks as well as rapid post-printing crosslinking. Moreover, low-viscosity gel inks are also incorporated with rheology modifier materials to enhance printability. For example, thermal-responsive methylcellulose (MC)-based materials are introduced to gelatin methacryloyl (GelMA) ink to increase viscosity and extend the thermal gelation window, allowing 3D bioprinting at a physiological temperature, thus improving cell viability [100]. Advanced network design by combining multiple crosslinking strategies can precisely control mechanical properties (e.g., stiffness, viscoelasticity) and physical properties (e.g., swelling, degradation). However, these intricate material–property relationships introduce additional optimization challenges.

Cells are often encapsulated in gel inks for tissue engineering and biomedical applications [61,101]. The density and size of cells have an impact on the crosslinking efficiency and rheological properties, as cells might behave as a physical hindrance between different interaction groups or chains in the bioink [102,103]. Additionally, cells may interfere with the chemical process of crosslinking reactions reducing the efficiency [104]. Certain crosslinking chemistries, such as thiol–ene, may involve functional groups which are naturally present on amino acids and therefore on the cell surface [105]. Moreover, the components (e.g., ions, amino acids) in the cell culture medium of cell suspension might be interacted with gel materials or buffering conditions used in the gel, compromising crosslinking and ink viscosity [106,107].

Multiple materials including a combination of hard materials and soft gels have been printed to create mechanically and biologically gradient structures [108,109,110]. However, layer delamination resulting from insufficient interfacial bonding or variations in crosslinking and mechanisms between layers might occur [111,112]. The introduction of an intermediate layer allowing bonding between different phases has been reported [113]. Moreover, ensuring homogeneity in composition and gelation kinetics across layers is essential to maintain mechanical integrity.

The performance of gel manufactured by additive manufacturing is a function of both the material characteristics and printing process variables. Depending on printing techniques, various parameters such as nozzle size [114], extrusion pressure [115], and printing speed [116] also affect printability. The wrong set of these conditions can result in printing failures including poor layer adhesion, filament collapse, and inhomogeneous structures [117]. Therefore, the printing parameters need to be carefully aligned with the rheological properties of the gel inks to ensure precise fabrication.

Smaller nozzles achieve finer structures but increase shear forces during printing [118,119]. This potentially damages cells and clogs the printing nozzle. Conversely, larger nozzles reduce shear stresses but compromise printing resolution. Embedded printing techniques, such as extruding gels into support baths, have been developed to mitigate this [120,121], while introducing additional complexities such as gel-support bath interactions. In addition, printing pressure must overcome the yield stress of gels to initiate flow. Higher concentrations of gel material (e.g., GelMA) facilitate cellular behavior while higher pressures generate damages on cells [99,122]. Printing speed further complicates the printing process. A slower printing speed improves filament deposition but increases the risk of nozzle clogging for fast-crosslinking systems (e.g., photocrosslinkable gels) [123]. Mismatches of the printing nozzle, pressure. and speed would result in poor printing, with either under-extrusion or over-extrusion.

Another essential printing quality factor is printing geometry. Path height, vertical offset between the printing nozzle and printing bed [124], must align with rheological properties such as elastic modulus. Larger path heights lead to stretching filaments and breakage [125]. Increased lag time between the gel leaving the nozzle and reaching the print bed would cause incorrect printing paths and incomplete corner structures [126]. However, smaller path heights result in nozzle interference and over-extrusion [124].

The multifactorial nature of gel additive manufacturing encompasses complex interdependencies between gel properties and printing process, making it challenging to optimize with conventional approaches [88]. Machine learning offers opportunities by mapping relationships across these conditions [52,83,124]. Unlike static systems, artificial intelligence (AI) enables adaptive real-time control with accurate prediction of the printing [17].

## 3. Machine Learning in Gels Material Design and 3D Printability

Machine learning enables the accurate prediction and optimization of fundamental gel properties such as rheological parameters [50], elastic modulus [51], creep dynamics [52], swelling ratio [127], permeability [128], and viscoelasticity [83,84,129]. Currently, no report mentions machine learning for biocompatibility and syneresis prediction. Neural networks and random forest models were used to predict the swelling behavior of temperature-responsive hydrogels based on synthesis parameters with high accuracy [130]. Deep learning approaches have been applied to forecast storage modulus G’ and loss modulus G’’ in polyacrylamide gels from composition and processing data [53]. In another study, random forest models linked shear viscosity to bioink composition and predicted cell viability in hydrogel formulations [131].

Decision tree and random forest algorithms excel in modeling nonlinear formulation property relationships [132]. One work [54] applied these methods to predict viscosity changes across shear rates and polymer ratios, achieving coefficients of determination above 0.98. Comparisons indicate that random forests often match or exceed neural networks in predicting rheological behavior when training sets are small. Support vector machines have been used to classify printable versus non-printable bioink formulations using rheological features [69].

Active learning combined with robotics advances autonomous gel discovery workflows. A peptide hydrogel study used Bayesian optimization with variational autoencoders to explore thousands of candidates and achieved higher hit rates in gelator identification [53,133]. Robotic experimentation systems integrated with random forest viscosity prediction enabled the closed-loop optimization of bioink formulations. Such intelligent systems are shifting gel formulation from intuition-based experimentation toward data-driven autonomous pipelines [70].

Data sparsity remains a challenge in gel ML applications. Many public hydrogel data repositories contain fewer than one thousand samples, limiting model generalization [134]. Purely data-driven models can produce nonphysical predictions when extrapolating beyond training boundaries. Physics-informed neural networks that impose viscosity and modulus constraints show promise for improved data efficiency and physical reliability [135].

### 3.1. Accelerating Material Discovery and Formulation Design

Machine learning has revolutionized the search for novel gel and hydrogel formulations with tailored properties [53,55,136,137,138,139]. By leveraging diverse ML algorithms—including neural networks, Gaussian Processes, extreme gradient boosting, random forests, multilayer perceptrons, and support vector regression—researchers have efficiently mapped the complex, multidimensional relationships among compositional variables, synthesis parameters, and targeted gel behaviors as shown in Table 1.

A notable example is the application of neural networks for predicting the molecular weight of collagen gels, enabling the precise tailoring of molecular architecture for biomedical uses [137]. Similarly, neural network models have been employed to improve gel stiffness in mildly refined yellow pea protein systems, streamlining the identification of optimal formulations for food and biomaterial applications [136]. For inorganic C–S–H (CaO–SiO_2_–H_2_O) gels, a combination of neural networks and Gaussian Process models successfully led to the design of compositions with significantly enhanced elastic moduli, outperforming traditional experimental methods in both efficiency and predictive power [138]. Extreme Gradient Boosting (XGB) has further enabled the accurate prediction of viscosity in polysaccharide colloids such as konjac glucomannan (KGM), facilitating rapid formulation screening for printable food gels and biopolymer materials [139].

Hybrid hydrogel systems have also benefited from ML optimization. For instance, HAMA/GelMA (hyaluronic acid methacrylate/gelatin methacrylate) hydrogels with tunable viscosity and improved printability have been developed using multilayer perceptron (MLP) and random forest (RF) models [55]. In polyacrylamide hydrogels, ML approaches such as MLP, variational autoencoders (VAE), and conditional VAE (CVAE) have enabled the simultaneous prediction of storage modulus (G′), loss modulus (G″), and formulation parameters, supporting both a forward and inverse design for mechanical property control [53]. In another case, logistic regression facilitated the efficient field screening of polyacrylamide gels crosslinked with organic agents, identifying promising candidates for oilfield and water shutoff applications [140].

Random forest algorithms have enabled the formulation of tunable, printable bioinks such as alginate/gelatin/TO-NFC (tempo-oxidized nanofibrillated cellulose) hydrogels by predicting viscosity and printability from compositional inputs [141]. For photodegradable acrylic and methacrylic gels, Bayesian optimization has driven the rapid development of new materials with fast, customizable degradation rates, critical for applications in tissue engineering and smart devices [142].

**Table 1:** New gel compositions designed by machine learning.

New Composition	Superior Properties	Machine Learning Algorithm	Ref.
Collagen gels	Predict molecular weight	Neural network	[137]
Mildly refined yellow pea ingredients	Improved gel stiffness	Neural network	[136]
CaO–SiO_2_–H_2_O C–S–H gels	Higher elastic moduli	Neural network, Gaussian Process (GP)	[138]
Polysaccharide colloids (KGM)	Better viscosity prediction	Extreme Gradient Boosting (XGB)	[139]
HAMA/GelMA hybrid hydrogels	Tunable viscosity	HydroThermo Multilayer Perceptron (MLP), Random Forest (RF)	[55]
Polyacrylamide hydrogels (PAA)	Predict G′, G″ or composition	Multilayer Perceptron (MLP), Variational Autoencoder (VAE), and Conditional Variational Autoencoder (CVAE)	[53]
Polyacrylamide (PAM) and organic crosslinkers	Better field screening	Logistic regression	[140]
Polysaccharide gels	Predict printability	SVR, Neural network, Convolutional neural network (CNN)	[50]
Atelocollagen, native collagen	Improved shape fidelity	Multiple regression	[51]
Hydrogel supercapacitor electrolytes	Higher capacitance, stability	SHAP (SHapley Additive exPlanations), tree models	[143]
Alginate/gelatin/TO-NFC bioink	Tunable viscosity, printable	Random forest	[141]
Photodegradable acrylic/methacrylic gels	Fast, tunable photodegradation	Bayesian optimization	[142]

**Figure 3:**
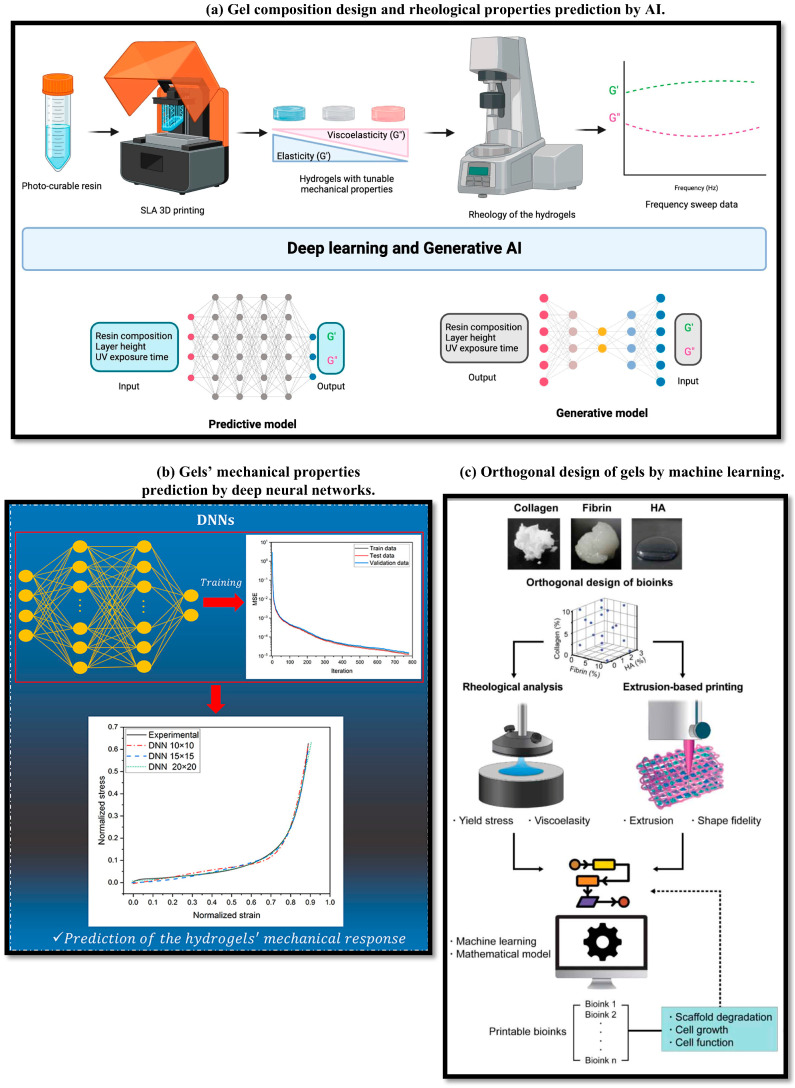
Workflow of artificial intelligence inform composition design. (**a**) This workflow uses deep learning and generative AI to both predict the rheological properties of 3D-printed hydrogels from resin composition and printing parameters, and inversely design printing recipes for targeted mechanical properties [53]. (**b**) The schematic combines experimental and cell studies of porous PVA/gelatin hydrogels with deep neural networks to accurately predict the mechanical response of the hydrogels based on material composition and microstructure [144]. (**c**) A strategy illustrates a bioink development strategy using a mathematical model and machine learning to predict and optimize 3D-printable bioink formulations based on their rheological properties for tissue engineering applications [51]. Panel (**a**) adapted with permission from ref. [53], MDPI, CC BY 4.0. Panel (**b**) adapted with permission from ref. [144], Elsevier. Panel (**c**) adapted with permission from ref. [51], IOP Publishing.

The integration of artificial intelligence and machine learning is fundamentally transforming how new gel formulations are designed, optimized, and validated for additive manufacturing. As illustrated in Figure 3, contemporary workflows utilize a combination of deep learning, generative modeling, and experimental feedback to predict critical rheological and mechanical properties from compositional and process variables, as well as to invert this mapping for autonomous recipe generation [51,53,144]. Figure 3a illustrates an advanced workflow where deep learning and generative artificial intelligence are harnessed to both predict and inversely design the rheological properties of 3D-printed hydrogels based on resin composition and printing parameters [53]. In this approach, comprehensive datasets encompassing various formulation recipes, process variables, and measured mechanical properties are used to train deep neural networks and generative models. The trained models can perform forward prediction—rapidly estimating properties such as viscosity, storage modulus, and printability from given material compositions. More importantly, the inverse design capability enables researchers to specify target mechanical or rheological properties and automatically generate candidate formulations predicted to meet these requirements, substantially accelerating the search for optimal recipes.

Figure 3b presents a schematic integrating experimental and cell studies of porous PVA/gelatin hydrogels with deep neural networks to accurately predict the compressive mechanical response of hydrogels, based on both material composition and microstructural descriptors [144]. In this workflow, experimental data—including microstructural images and quantitative metrics describing porosity, crosslinking density, and pore architecture—are fed as inputs into supervised learning algorithms. These neural networks are trained to map the microstructural and compositional inputs to macroscale mechanical properties such as compressive strength and modulus. By capturing the intricate structure–property relationships that govern gel mechanics, this approach allows for the rational tuning of gel formulations for targeted mechanical performance. Figure 3c highlights a workflow for bioink development in which mathematical modeling and machine learning are combined to predict and optimize 3D-printable formulations based on rheological measurements and target printability metrics [51]. Here, data from rheological tests—such as viscosity, elastic modulus, and yield stress—are used as input features for machine learning models, which are trained to classify or score the printability of candidate bioinks. These models guide the iterative formulation process by suggesting compositions with an increased probability of successful 3D printing, significantly reducing the reliance on trial-and-error experimentation. Such predictive frameworks have enabled the rapid identification of bioink formulations that not only meet the required mechanical and flow properties but also maintain biocompatibility and cellular viability, advancing the clinical translation of 3D bioprinted tissues.

### 3.2. Enhancing Gels’ 3D Printability

The integration of machine learning (ML) into gel-based additive manufacturing has fundamentally reshaped the prediction and optimization of 3D printability, allowing for unprecedented control over material formulation and process parameters. Printability, which encompasses the ease of extrusion, shape retention, and fidelity of printed structures, depends on a complex interplay of rheological, mechanical, thermal, and formulation-related properties [145]. By leveraging large datasets of compositional and processing parameters, ML algorithms such as neural networks, random forests, and support vector machines can accurately predict fundamental material properties, including the viscosity, elastic modulus, swelling ratio, and viscoelasticity [55,137,139], in Table 2. Instead of laboriously synthesizing and testing every candidate formulation, researchers can now use data-driven models to efficiently screen vast compositional spaces, identifying promising candidates with minimal experimental effort [45]. For example, deep learning models have been used to predict the rheological behavior and printability of bioinks and food gels, substantially reducing the number of required experiments [50,53]. Generative models and active learning further enable the autonomous design of new gel recipes optimized for target properties [133].

Central to printability prediction are rheological properties, such as viscosity, storage modulus (G′), loss modulus (G″), shear-thinning behavior, and yield stress [50,55,137,139,146]. These properties govern both the flow of gels through the print nozzle and the fidelity of shape after deposition. Machine learning models have demonstrated robust predictive power for these parameters, with random forest and gradient boosting methods excelling at viscosity and modulus prediction using compositional and processing data [50,55,139,146]. Deep learning approaches, including multilayer perceptrons and variational autoencoders, have been particularly effective in mapping complex nonlinear dependencies between molecular composition, printing parameters, and resultant mechanical or rheological properties [53,133,135,147]. Physics-guided neural networks further enhance model reliability and generalizability, incorporating physical constraints into data-driven predictions [135].

Mechanical properties, such as elastic modulus, Young’s modulus, gel strength, and crosslinking potential, are equally vital for maintaining structural integrity post-printing [50,51,55,136,138,140]. Gaussian process regression, tree-based models, and neural networks have been used to quantify these parameters and correlate them to successful printing outcomes [142,144,146]. For instance, studies applying support vector regression and SHapley Additive exPlanations (SHAP) have elucidated the most influential factors determining whether a given hydrogel formulation will print successfully or fail [146]. Surface tension and stiffness, key for stacking layers and achieving desired construct geometry, have also been incorporated into predictive frameworks using both neural and ensemble methods [55,138].

Beyond classical rheological and mechanical factors, thermal sensitivity—such as denaturation temperature, gel point, and maximum process temperature—directly impacts the window of printable conditions and material stability during processing [137,146,147]. Additionally, ML approaches are being used to predict water holding capacity and optimize formulation parameters, including concentration, molecular weight, and water content, to modulate gel strength and viscosity precisely [53,55,136,138,139].

## 4. AI-Driven Process Optimization in Gel Additive Manufacturing

**Figure 4:**
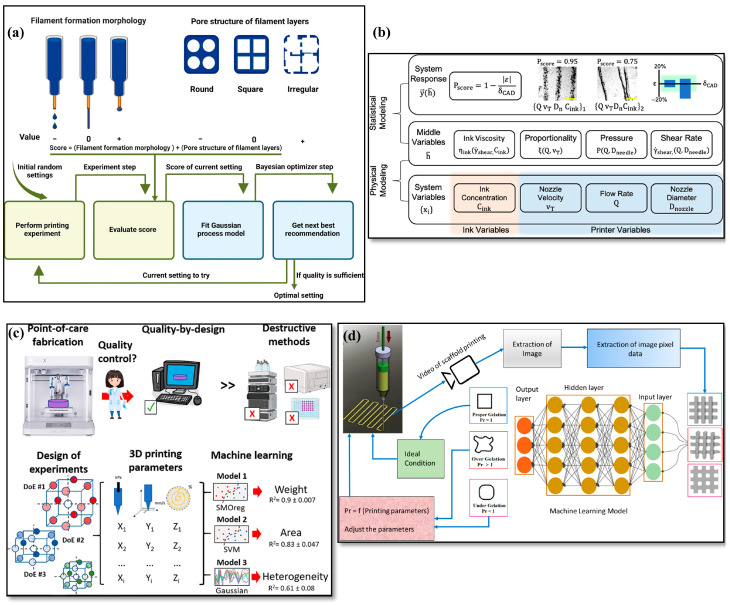
Process optimization via machine learning. (**a**) The scoring system for evaluating bioink printability based on printed layer morphology and pore structure, with optimal printability [149]. (**b**) The hierarchical machine learning (HML) model structure, showing how experimental print variables are connected through a middle layer of physical parameters to predict 3D print fidelity [150]. (**c**) Machine learning enables non-destructive, quality-by-design optimization of 3D-printed hydrogel properties for biomedical use [151]. (**d**) The workflow of computer vision and machine learning is used for real-time, in situ monitoring and adjustment of 3D bioprinting to ensure optimal printability by detecting gelation conditions during the process [152]. Panel (**a**) adapted with permission from ref. [149], Elsevier. Panel (**b**) adapted with permission from ref. [150], American Chemical Society. Panel (**c**) adapted with permission from ref. [151], Elsevier. Panel (**d**) adapted from ref. [152], Elsevier, CC BY-NC-ND 4.0.

In this section, we discuss machine learning driven additive manufacturing process optimization. Figure 4 serves as a preview and workflow on how to modify the process by machine learning. One critical approach illustrated (Figure 4a) is the use of scoring systems to quantitatively evaluate bio-ink printability based on printed layer morphology and pore structure, enabling the selection of optimal formulations and print conditions [149]. Such systems minimize subjective judgment and improve reproducibility by providing clear criteria for assessing print quality.

Another important strategy (Figure 4b) involves hierarchical machine learning models that link experimental print variables with physical parameters to accurately predict three-dimensional print fidelity [150]. This intermediate step allows for more interpretable and robust predictions, as it connects directly measurable process variables to the underlying material behavior. These hierarchical models can optimize both the process and the resulting material properties, facilitating high-fidelity printing with fewer trial-and-error experiments.

In Figure 4c, machine learning is also enabling non-destructive and quality-by-design optimization of hydrogel properties specifically for biomedical applications. Through predictive modeling, key process and formulation parameters can be tuned to yield desired mechanical and biological outcomes, reducing reliance on destructive testing and lengthy validation protocols [151]. By leveraging these models, researchers can accelerate the translation of printed hydrogels into clinical use.

Real-time process control is further enhanced through the integration of computer vision and machine learning as shown in Figure 4d. In situ monitoring systems can detect changes in gelation conditions or identify print defects as they occur, allowing immediate adjustments to printing parameters [152]. This closed-loop feedback not only ensures consistent print quality but also improves process robustness and scalability. As a result, the combination of machine learning with advanced sensing technologies is paving the way toward fully autonomous, intelligent bioprinting systems.

### 4.1. Reasons to Use Machine Learning

Additive manufacturing (AM) of gels holds enormous promise for applications in tissue engineering, soft robotics, electronics, and drug delivery, due to the inherent versatility and tunability of these soft materials [40]. However, traditional experimental approaches to optimizing material composition and process parameters are labor-intensive and reliant on trial and error, often resulting in suboptimal performance and limited reproducibility [14]. The adoption of machine learning (ML) in gel-based AM is rapidly transforming the field by enabling accelerated material discovery [16], predictive process optimization [17], real-time quality control [153], and intelligent automation [154].

By enabling the efficient exploration of material and process spaces, machine learning drastically reduces the number of failed experiments and the consumption of costly or rare reagents [155]. Predictive modeling also helps minimize material waste and energy consumption, supporting the development of greener and more sustainable additive manufacturing workflows [156].

Classical machine learning models such as decision trees, random forests, and linear or logistic regression are widely regarded as the most interpretable approaches for regulatory purposes [157,158], particularly in gel printing [28]. These models offer straightforward insights into how input features influence predictions, with clear decision paths or feature importance rankings that facilitate transparency and traceability—qualities highly valued by regulatory bodies. In contrast, complex models such as deep neural networks and ensemble methods often function as “black boxes”, making it more challenging to directly understand the rationale behind their outputs. However, the interpretability of these advanced models can be enhanced through the application of post hoc explanation tools such as SHAP (SHapley Additive exPlanations) [159], which help elucidate feature contributions and decision mechanisms.

### 4.2. Wide Availability of Resources

The growth of publicly available datasets, open-source ML packages, and standardized data formats has greatly facilitated the application of ML to gel-based AM as shown in Table 3. Comprehensive repositories such as Mendeley [160], Zenodo [161], Figshare [162], and NIST [163] provide a rich resource for model training, benchmarking, and cross-study comparison, accelerating progress in the field.

A diverse array of open-source datasets and machine learning packages have significantly propelled research and innovation in gels additive manufacturing process optimization. Among publicly accessible datasets, repositories such as Mendeley, Google Dataset Search, NIST, Zenodo, Figshare, and the AmeriGEOSS Community Platform DataHub have been instrumental in supporting data-driven advances in this field [160,161,162,163,164]. Mendeley and Zenodo, in particular, offer not only a breadth of datasets but also journal articles, formulations, and benchmarking resources, making them some of the most frequently referenced platforms. The adoption of open-source packages has similarly accelerated the implementation of machine learning algorithms, with Scikit-learn standing out as the most commonly used toolkit for classical machine learning models such as support vector machines, decision trees, random forests, and gradient boosting, which have enabled applications ranging from shrinkage prediction and rheological property modeling to high-fidelity printability optimization [51,149,150,165,166]. Meanwhile, TensorFlow and PyTorch 2.3.0 dominate deep learning development, supporting neural network-based approaches for tasks such as printing speed optimization, real-time video monitoring, scaffold quality prediction, and light scattering compensation [151,153,167,168]. Keras is frequently favored for rapid prototyping of deep learning models, especially for extrusion pressure and structural conformity prediction [169]. The synergy between these open-source tools and expansive data repositories has enabled not only predictive modeling and process optimization but also the simultaneous tuning of formulation, material, and process variables, thereby reducing experimental burden and enhancing reproducibility [51,149,150,170]. Collectively, the prevalence of Scikit-learn across a multitude of studies underscores its pivotal role, while Mendeley and Zenodo emerge as the most frequently utilized data platforms, facilitating cross-study benchmarking and accelerating methodological development.

**Table 3:** Open-source packages, machine learning models, datasets and their applications cases in gels additive manufacturing process optimization.

Machine Learning		Description and Features	Application
Open-source datasets	Mendeley [160]	Available from https://data.mendeley.com (accessed on 1 June 2025)	Datasets [171,172,173,174,175]
	Google Dataset search	Available from https://datasetsearch.research.google.com (accessed on 1 June 2025)	Datasets [176,177]
	NIST [163]	Available from https://data.nist.gov (accessed on 1 June 2025)	Datasets [178]
	Zenodo [161]	Available from https://zenodo.org (accessed on 1 June 2025)	Datasets, journal papers, and formulations [179,180,181,182,183,184,185,186,187,188,189,190,191,192,193,194,195,196,197,198,199,200,201,202,203,204,205,206,207,208,209,210,211]
	Figshare [162]	Available from https://figshare.com (accessed on 1 June 2025)	Figures, videos, and datasets [212,213,214,215,216,217,218]
	AmeriGEOSS Community Platform DataHub [164]	Available from https://data.amerigeoss.org (accessed on 1 June 2025)	Patents, datasets, and project reports [219,220]
Open-source packages	Scikit-learn [221]	Classical machine learning models (easy-to-use, general-purpose). Typical models: Support Vector Machine, Decision Tree, Random Forest, Logistic Regression, k-Nearest Neighbors, Principal Component Analysis, k-Means Clustering, Gradient Boosting Machine, Extreme Gradient Boosting (XGB), PCA.	Shrinkage [222], gel point [223], gelatin, pore size and stiffness [170], rheological properties [224], high elastic modulus and yield stress [51], simultaneously optimize material, formulation, and processing variables [165], high-fidelity [150], shear rate [225], compressive modulus, density, and porosity [226], printability from rheological measurements [166], high viscosity [149], storage and loss moduli, and hardness for extraordinary printability [227]
	TensorFlow [228]	Deep learning and neural networks (high flexibility for research and production). Typical models: Convolutional Neural Network, Recurrent Neural Network, Deep Neural Network, Generative Adversarial Network, Long Short-Term Memory Network, Transformer Model.	Printing speed, printing pressure and infill percentage [151], and real-time videos monitoring [153,167]
	PyTorch [229]	Research, flexible deep learning model development, Convolutional Neural Network, Recurrent Neural Network, Transformer Model, Diffusion Model.	Light scattering compensation [168], monomer composition ratios [230], printability and scaffold quality [231], and material deposition temperature monitoring [154]
	Keras [232]	High-level API; excellent for quick development of deep learning models (uses TensorFlow backend). Convolutional Neural Network, Recurrent Neural Network, Deep Neural Network, Long Short-Term Memory Network.	Minimum extrusion pressure (MEP) and printed structure conformity (PSC) [169]

### 4.3. Predictive Process Optimization

One of the major advantages of integrating ML in gel AM is the ability to predict the effect of process parameters—such as nozzle diameter, extrusion pressure, print speed, and temperature—on print fidelity and final material properties in Table 4. Classical ML algorithms (e.g., decision trees [233], random forests [132], support vector machines [234]) and advanced deep learning architectures like CNN [235] and the deep neural network [236] have demonstrated high accuracy in modeling the complex, nonlinear relationships between input parameters and print outcomes [69,138]. These models enable rapid optimization and parameter tuning, minimizing the cost and time associated with random experimental trials and reducing material waste [31,38,51,55]. Furthermore, multi-response optimization methods can simultaneously consider multiple printability metrics, facilitating the development of robust, reproducible processes [124,140].

### 4.4. Real-Time Quality Control and Autonomous Process Control

Real-time monitoring in gel-based additive manufacturing, particularly hydrogel extrusion processes, is essential to ensure precise structure deposition and mechanical performance. Traditional optical imaging techniques such as optical coherence tomography face limitations due to gel opacity and high water content, which reduce imaging depth and accuracy during printing [241,242]. The inherent variability in gel rheology, nozzle–substrate interactions, and post-processing crosslinking dynamics introduce additional quality control challenges unique to hydrogel-based systems.

A recent study introduced a real-time, in situ ultrasound monitoring system integrated into a Bio X bioprinter platform. By capturing ultrasound reflections from alginate–gelatin hydrogel layers, the system identifies subtle defects such as layer delamination, nozzle scraping, and gravitational sagging, with subwavelength resolution (~0.78 λ) [241]. This method enables a layer-by-layer quality assessment, real-time parameter adjustment, and optimal crosslinking time determination in CaCl_2_ baths.

Machine vision systems have also been tailored for gel-based processes. For instance, computer vision systems monitor hydrogel printing with embedded fibers to prevent misalignment in real time by adjusting extruder movement and fiber feed dynamically [243,244,245,246]. While originally demonstrated in composite filament deposition, these principles are transferable to hydrogel printing, enabling closed-loop control.

Machine learning, particularly in combination with computer vision, is enabling real-time, in situ monitoring of gel 3D printing processes [50,166,247]. Convolutional neural networks and other image-based ML approaches can rapidly detect defects, misprints, or suboptimal gelation conditions during printing, triggering immediate corrective actions [167,237,248,249]. Such closed-loop systems help ensure consistent print quality and reproducibility, even in the face of batch-to-batch variations in materials or environmental changes. Robotic experimentation systems, coupled with ML-based feedback, are moving the field toward autonomous and intelligent manufacturing pipelines [250,251].

Despite rapid advancements in real-time monitoring and autonomous control in gel additive manufacturing, several significant challenges remain. The challenge involves the complexity and volume of process data generated during real-time monitoring. High-speed imaging, ultrasound signals, rheological data, and other sensor outputs quickly result in large, multimodal datasets. Effectively fusing and interpreting these data streams in real time requires sophisticated data processing pipelines and often machine learning models that can operate efficiently with minimal latency [252]. However, ensuring interoperability across different platforms, maintaining data integrity, and dealing with inconsistent formats or sensor drift remain unresolved problems [253]. Additionally, the development and deployment of standardized data protocols for gel additive manufacturing are still in their infancy, impeding seamless cross-study comparison and reproducibility [242]. The generalization and robustness of machine learning models represent further hurdles. Most ML algorithms and neural networks used for process control are trained on datasets specific to particular gel formulations, bioprinter models, or environmental conditions. When these models are applied to new materials, different crosslinking chemistries, or altered hardware setups, their performance can degrade rapidly, necessitating continual retraining and calibration [151,231]. Another challenge is the integration of autonomous control algorithms with existing hardware and process workflows. Many gel printing systems were not originally designed for closed-loop or adaptive feedback operation. Retrofitting these platforms to accommodate real-time control, ensuring mechanical stability, and validating control actions in compliance with safety and quality standards—especially for biomedical applications—are nontrivial tasks [254,255,256]. Furthermore, maintaining biocompatibility and sterility during automated interventions, particularly for cell-laden hydrogels, requires additional consideration [257].

## 5. Research Limitations and Outlook

### 5.1. Limitations

Despite recent progress, several technical and translational barriers hinder the maturity of gel printing. First, print resolution and structural fidelity remain constrained by the inherently low viscosity of most gels, leading to collapse or deformation in overhanging regions and complex architectures [258]. Coupled with this is the lack of standardized rheological benchmarks: while printability is often qualitatively assessed, the absence of unified metrics makes cross-study comparison challenging. Moreover, real-time monitoring in gel printing lags behind polymer AM. Sensor techniques such as optical coherence tomography and ultrasound have been explored, but have yet to achieve the robustness required for in-process defect detection, especially under biologically relevant conditions [259].

From a materials design standpoint, there is limited integration of data-driven formulation pipelines. Existing studies using the design of experiments and machine learning have focused predominantly on food-grade or simple biomaterial gels, leaving robust organogel systems for structural or functional use less explored [258]. Furthermore, while responsive and 4D hydrogels show promise in tissue modeling and soft robotics, current models lack predictive accuracy due to incomplete incorporation of complex thermomechanical or swelling dynamics [14,245,260]. Scalability and standardization remain significant hurdles: most gel-printed constructs are small-scale and lack quality benchmarks—clinical or industrial translation requires larger constructs, repeatable processes, and regulatory-aligned validation protocols.

### 5.2. Outlook

As gel-based printing (particularly hydrogels, organogels, and bioinks) continues to advance, its convergence with machine learning and materials innovation presents both exciting opportunities and challenges. Recent reviews highlight several crucial future directions.

#### 5.2.1. Real-Time Defect Detection with Multimodal Monitoring

Real-time defect detection with multimodal monitoring holds immense potential to revolutionize gel-based additive manufacturing, especially as new combinations of ultrasound, computer vision, and machine learning-based anomaly detection become standard. For example, ultrasound tracking of alginate-gelatin prints achieved millimeter-scale detection of interlayer bonding and deformation in near real-time [241]. In the future, multimodal systems could enable continuous, in-process optimization during the fabrication of complex biomedical scaffolds, ensuring uniformity and structural integrity in patient-specific implants. For tissue engineering, integrating real-time imaging and ML can facilitate automated correction of defects or incomplete layers during the printing of cell-laden hydrogels, improving construct viability and clinical reliability. In soft robotics and wearable devices, such monitoring will allow precise tuning of mechanical gradients and embedded functions as printing progresses, supporting the fabrication of multi-material and multi-functional components. Beyond healthcare, real-time multimodal monitoring can advance food printing and soft electronics by enabling closed-loop control for consistent texture, patterning, and functionality, even under variable environmental conditions. Ultimately, the convergence of these technologies is set to drive fully autonomous, intelligent gel printing systems capable of self-correcting and delivering reliable, high-performance products for a diverse set of emerging applications.

#### 5.2.2. Predictive Stimuli-Responsive 4D Gel Systems

The combination of machine learning and predictive modeling is set to unlock transformative applications for stimuli-responsive 4D gel systems. By training models on datasets that connect gel composition, structure, and environmental cues to dynamic behaviors, researchers will be able to design gels that perform highly specific shape changes or force outputs under precise conditions. In the future, these predictive 4D gel systems could be used to create programmable scaffolds for tissue engineering. In soft robotics, gels with machine-learned deformation profiles will enable devices that adapt their motion, stiffness, or gripping ability on demand, leading to safer and more versatile interaction with biological tissues or delicate objects [261,262]. In drug delivery, 4D gels could provide temporally and spatially controlled release of therapeutics, responding intelligently to physiological signals such as pH, temperature, or enzymatic activity. Moreover, in areas like bioelectronics and responsive surfaces, ML-optimized gels may enable adaptive interfaces that change conductivity, permeability, or optical properties in real time. Ultimately, as datasets grow and predictive models become more robust, stimuli-responsive 4D gel printing guided by machine learning will drive innovation across biomedicine, soft robotics, sensing, and smart materials, creating adaptive systems that respond seamlessly to complex and changing environments.

#### 5.2.3. AI Assisted In-Body Gel Printing

A recent study demonstrated the ultrasound-guided printing of drug-laden hydrogels inside a mouse bladder, enabling localized chemotherapy with real-time shape control [263]. For future possibilities, the integration of machine learning with in-body gel printing using ultrasound-responsive bioinks is poised to transform minimally invasive medicine. As deep learning and adaptive control algorithms become more sophisticated, they will enable real-time interpretation of ultrasound images, guiding the precise formation and placement of gels within dynamic living tissues. Machine learning will also facilitate the adaptive adjustment of printing parameters in response to individual anatomical and physiological variations, supporting the delivery of patient-specific therapies.

## Figures and Tables

**Table 2:** Machine learning predictions in gels’ 3D printability.

Property Type	Key Feature	Effect on Printability	Machine Learning Methods
Rheological	Viscosity [50,55,137,139,146]	Determines ease of extrusion	Random forest, SHAP; gradient boosting
	Storage modulus (G′), loss modulus (G″) [53,137,139,146,147]	Determines viscoelastic behavior and shape retention	Python 3.9 libraries (NumPy, Pandas, Scipy, and Sklearn) used for curve fitting, smoothing, and extrapolation for G′ and G″ curves; MLP (Multilayer Perceptron), VAE (Variational Autoencoder)
	Shear-thinning [50,55]	Enables smooth extrusion	Random forest
	Yield stress [50,55], critical stress [146]	Supports shape after printing and maintains printed shape	SHAP feature ranking; support vector machines
	Angular frequency (ω) [139]	Key parameters affecting viscosity	Decision tree (DT), random forest (RF), gradient boosting decision tree (GBDT), extreme gradient boosting (XGBoost)
Mechanical	Elastic modulus [50,55]	Structural support post-printing and ensures structural integrity	Regression models; decision tree
	Crosslinking potential [50]	Long-term stability	Data-driven formulation selection
	Surface tension [55]	Affects layer stacking	Neural networks
	Stiffness components [138]	Quantifies gel mechanical properties	Gaussian Process (GP) Regression, Neural Network (NN)
	Young’s modulus (Eu, kPa) [136]	Measures gel stiffness	Neural Network
	Gel strength (g × mm) [148]	Indicates firmness, quality	Long short-term memory network, Convolutional Neural Network
Thermal Sensitivity	Denaturation temperature (Td) [137], gel point [147], max process temperature [146]	Sets upper limit for printing temperature to avoid degradation	SVM, random forest, extreme gradient boosting (XGB)
	Water holding capacity (WHC) [146]	Influences structure formation	Random forest, decision tree
Formulation	Concentration [53,55,136,139,146], molecular weight (MW) [139], H_2_O content (molar %) [138]	Modifies gel strength, key parameters affecting viscosity and mechanical properties	Logistic regression, decision tree, Neural Network

**Table 4:** Machine learning driven process optimization in gels additive manufacturing.

AM Methods	AM Process Parameter	How to Improve the Process	Machine Learning Methods	Ref
Extrusion bioprinting	Nozzle size, pressure	Optimize alginate formulation	Deep learning	[237]
	Bioink composition	Control hydrogel rheology	Physics-informed ML	[147]
	NHS/EDC concentrations	Adjust crosslinking for flexibility	XGBoost, SHAP	[238]
	Nozzle speed, diameter	Improve viscosity, digestibility	ANN-GA, RSM	[149]
	Flow rate, nozzle design	Reduce trial-and-error	Decision trees	[239]
	Layer height, print speed	Bioink optimization	Multiscale ML, Big Data	[239]
	Bioink rheology	Multi-response optimization of printability	ANN, DOE, RSM	[169]
	Printing defects	Detect in real time, reduce waste	CNN, deep learning	[153]
	Ink composition, nozzle	Suggest ink formula and print settings	Bayesian optimization	[149]
	Bioink comp., shear rate	Predict viscosity, optimize formula	Random forest, DT, PF	[141]
	ADA-GEL, pore size	Tune stiffness for tissue engineering	XGBoost	[170]
	Print speed, flow rate, and nozzle, ink	Predict high-fidelity hydrogel prints	Hierarchical ML	[150]
	Bioink comp., temp, speed, and pressure	Minimize trial-and-error for printability	Bayesian optimization	[240]
	Path height, nozzle temp, and composition	Maximize print fidelity, minimize tests	SVM	[124]
Vat photopolymerization	Monomer composition	Targeted property selection	Active learning, ML regression	[230]
Food printing	Starch/protein ratio	Predict printability and texture	PCA, SVM	[227]

## Data Availability

The original contributions presented in this study are included in the article. Further inquiries can be directed to the corresponding author.
